# Identifying tumor antigens and immune subtypes of renal cell carcinoma for immunotherapy development

**DOI:** 10.3389/fimmu.2022.1037808

**Published:** 2022-11-03

**Authors:** Xinglin Chen, Tongtong Zhang, Xinyu Zhai, Zhong Wan, Minyao Ge, Chengzong Liu, Mingyue Tan, Dongliang Xu

**Affiliations:** Urology Centre, Shuguang Hospital Affiliated to Shanghai University of Traditional Chinese Medicine, Shanghai, China

**Keywords:** DBH-AS1, immunotherapy, mRNA vaccine, renal cell carcinoma, immune subtypes

## Abstract

Renal cell carcinoma (RCC) is one of the leading causes of death in men. Messenger ribonucleic acid (mRNA) vaccines may be an attractive means to achieve satisfactory results. Cancer immunotherapy is a promising cancer treatment strategy. However, immunotherapy is not widely used in renal cell carcinoma, as only a few patients show a positive response. The present study aimed to identify potential antigens associated with renal cell carcinoma to develop an anti-renal cell carcinoma mRNA vaccine. Moreover, the immune subtypes of renal cell carcinoma cells were determined. The Cancer Genome Atlas (TCGA) analysis revealed gene expression profiles and clinical information. Antigen-presenting cells infiltrated the immune system using Tumor Immune Estimation Resource (TIMER) tool (http://timer.cistrome.org/). GDSC (Genomics of Drug Sensitivity in Cancer) database were used to estimate drug sensitivity. The 13 immune-related genes discovery could be targets for immunotherapy in renal cell carcinoma patients, as they were associated with a better prognosis and a higher level of antigen-presenting cells. These immune subtypes have significant relationships with immunological checkpoints, immunogenic cell death regulators, and RCC prognostic variables. Furthermore, DBH-AS1 was identified as a potential antigen for developing an mRNA vaccine. The CCK8 assay demonstrated that the proliferative capacity of 786-O and Caki-1 cells overexpressing DBH-AS1 was higher than in the control group. In addition, transwell assay revealed that 786-O and Caki-1 cells overexpressing DBH-AS1 showed higher invasion capacity compared with control. This study provides a theoretical basis for the development of mRNA vaccines. Our findings suggest that DBH-AS1 could be potential antigens for developing RCC mRNA vaccines.

## Introduction

Men are more likely than women to develop renal cell carcinoma (RCC), which is one of the world’s 10 most common types of cancer (1). Most deaths from renal cell carcinomas are caused by clear cell renal cell carcinomas (ccRCCs). The treatment depends on the diagnosis of tumor characteristics and staging. Currently, most patients with RCC present with metastatic disease, and a 30%–40% chance of developing metastases is expected in the remaining patients (2). There are many places where metastatic RCC can occur, such as the lungs, lymph nodes, bones, liver, and brain. RCC has a broad metastatic potential and is very common even after radical nephrectomy (3). According to literature reports, most metastatic lesions occur within the first 5 years (4). A previous study reported that age, race, and family history were significant risk factors for RCC patients (5).

Cancer immunotherapy can be divided into passive immunotherapy and active immunotherapy (6). Active immunotherapy stimulates a patient’s immune system to activate natural killer cells or cytotoxic T cells or to produce antibodies against tumor-specific antigens (7). One of the functions of immune checkpoint inhibitors is to block programmed death 1 (PD-1), restoring T cells to target cancer cells (8). Various drugs have been developed to inhibit certain solid tumor progression. Nivolumab and pembrolizumab are emerging antitumor drugs that work by blocking the immune checkpoint protein PD-1, hence promoting T-cell recovery to target cancer cells. Furthermore, PD-L1 inhibitors are currently used in some cancers and are being explored for other cancers (9).

In the last two decades, treating and managing metastatic RCC have undergone substantial improvements. Initially, cytokines were utilized in first-generation immunotherapies. Interleukins or interferons were the standard approaches, with poor results (10). Immune checkpoint inhibitors (ICIs) alone or in combination have shown better results than traditional immunosuppressants (11). Targeted immunotherapy can be used in place of antiangiogenic drugs because ccRCC is also considered an immunogenic tumor with many immune cells like tumor-infiltrating lymphocytes (TILs) (12). The tumor microenvironment (TME) is complex and evolving. In addition to stromal cells, fibroblasts, and endothelial cells, the TME also includes innate and adaptive immune cells. TME plays a critical role in drug resistance, according to studies combining antiangiogenic and targeted immunotherapies, which are currently available as a first-line treatment option (13). It is possible to develop resistance to ICIs through primary, congenital, secondary, or acquired mechanisms. These are neoantigen loss, deficient antigen presentation, alternative immune checkpoints, and deficient interferon signaling. Interferon-γ is key in enhancing the PD-L1 expression and inducing immunosuppressive molecules (14). TIGIT, LAG-3, TIM-3, and other immune checkpoints suppress antitumor immunity, contributing to drug resistance. These resistance mechanisms are being overcome with new treatments currently being evaluated in ongoing clinical trials. Identifying biomarkers for metastatic kidney cancer is critical to select better treatments, lowering costs, and improving survival (15). Nevertheless, the limitations of the most studied biomarkers, PD-L1 immunohistochemistry, and TMB make it imperative to find more robust markers. New technologies may be able to provide this assistance.

The current study aimed to explore new RCC antigens, provide a basis for developing new immunotherapy drugs, and define immune subtypes that can be used to improve immunotherapy response in RCC patients. Furthermore, the study sought to provide insights into targeted therapy in RCC by conducting correlation analysis of antigen-presenting cells, prognostic-related genes, immune subtype analysis, immune checkpoints, and immune modulators.

## Methods

### Data sources

A key feature of The Cancer Genome Atlas (TCGA) database (https://portal.gdc.cancer.gov/) is its representation of cancer gene expression, miRNA expression, copy number variation, DNA methylation, and single nucleotide polymorphisms (SNPs). TCGA database was used to retrieve mRNA expression data of the Kidney Renal Clear Cell Carcinoma (KIRC) cohort. cBioPortal (http://www.cbioportal.org,v3.2.11) provides open access to raw data from large-scale genome studies. In this study, cBioPortal was used to explore potential anti-tumor antigen gene changes in TCGA cohort. TCGA-KIRC on cBioPortal was used to retrieve complete expression profiles and survival information, and ImmPort retrieves immune-related genes for each patient.

### Tumor Immune Estimation Resource analysis

An advanced tool for systematically analyzing immune infiltration in different cancer types is called TIMER (https://cistrome.shinyapps.io/Timer/). A TIMER analysis was conducted to examine the relationship between immune cell infiltration and the expression level of the identified effective antigen.

### Detection of the differential expression genes

We screened differentially expressed genes using the edgeR filter criteria [log2|fold change| > 2, false discovery rate (FDR) < 0.05]. The volcano plot displayed filtered results, as red indicates genes that have been upregulated, and blue indicates genes that have been downregulated.

### Classification of immune subtypes

We quantified individual scores for each tumor case using the single-sample gene set enrichment analysis (ssGSEA) method. ssGSEA computes overexpression measures for a list of genes of interest relative to all other genes in the genome using a rank-based method. ssGSEA scores were calculated using log-transformed RNA-seq or microarray data. Our next step was to classify RCCs according to 29 immunobio signature enrichment levels (ssGSEA scores) and examine both tumor purity and the immune score for each RCC.

### Drug sensitivity analysis

According to the Genomics of Drug Sensitivity in Cancer (GDSC) database, the chemotherapy sensitivity of each tumor sample was predicted with “pRRophetic” R package (https://www.cancerrxgene.org/). IC_50_ values for each chemotherapy drug were further determined by regression analysis. We performed cross-validation on thGDSC training set 1e 0 times to test the regression and prediction accuracy. Each parameter was set to its default value, including the “combat” parameter, which removes batch effects and averages repeated gene expressions.

### Immune cell infiltration analysis

The relative proportions of 22 immune infiltrating cells were determined by analyzing RNA-seq data from RCC patients in different sub-groups using CIBERSORT algorithm. Following this, Spearman correlation analysis was performed to determine how gene expression relates to immune cell infiltration. It was considered statistically significant when p-value <0.05.

### Gene set variation analysis

Gene set enrichment was evaluated using gene set variation analysis (GSVA), a non-parametric, unsupervised method. The gene-level changes in this analysis were transformed into pathway-level changes by scoring the gene set of interest, followed by determining the sample’s biological function. The molecular signatures database (version 7.0) was used to retrieve the gene sets in the present study. A comprehensive evaluation of potential biological function changes in various samples was then conducted using the GSVA algorithm.

### Construction of the prognostic prediction model

The univariate Cox regression, multivariate Cox regression, and LASSO regression were used to investigate the genes closely associated with RCC prognosis.

### Weighted correlation network analysis

Weighted gene coexpression networks were constructed to evaluate co-expressed gene modules, genotype–phenotype relationships, and core genes in the network. Using weighted correlation network analysis (WGCNA) R package, we constructed a coexpression network using all genes in the dataset. We further analyzed genes with the highest variances using 3 as a threshold. Furthermore, a network connectivity estimation was conducted by transforming the weighted adjacency matrix into a topological overlap matrix (TOM). A hierarchical clustering tree was also constructed based on TOM matrix using hierarchical clustering. Different branches of the clustering tree represented different gene modules, whereas different colors represented different gene modules. A module was created by grouping genes whose expression patterns are similar. The weighted correlation coefficients of thousands of genes allowed the identification of multiple modules based on gene expression patterns.

### GO and KEGG enrichment analysis

Key genes were annotated, and candidate genes’ functions were explored using ClusterProfiler package. We explored related functional categories using gene ontology (GO) and Kyoto Encyclopedia of Genes and Genomes (KEGG). In GO and KEGG enrichment pathways, statistical significance was determined by p-values and q-values <0.05.

### Gene set enrichment analysis

MSigDB (http://www.gsea-msigdb.org/gsea/downloads.jsp) was used to retrieve gene sets. GSEA was performed on the gene sets to identify enriched GO terms and KEGG pathways. The 50 best terms were selected from each subtype based on their significance.

### Cell culture

786-O and Caki-1 cells were conducted in 1640 and McCoys 5A media, respectively. 1640 and McCoys 5A media are rich in 10% fetal bovine serum, 1% penicillin-streptomycin, and 1% glutamine in a humidified atmosphere with 5% CO_2_ at 37°C. Overexpression plasmids and control vectors were designed and constructed by Shanghai Jikai Gene Co, Ltd. Transfection of the expression plasmid was performed according to the manufacturer’s instructions.

### CCK8 assay

CCK8 assay was conducted using the CCK8 kit (Dojindo, Shanghai, China). Briefly, 1,000 cells were planted into each well with 100 μl medium, and 10 μl of CCK8 was added to each well. After incubation at 37°C for 24 h in a humidified incubator with 5% CO_2_, the proliferative ability of the cells was measured at 450 nm.

### Transwell assay

Transwell chambers with pore sizes of 8.0 mm were added to a 24-well plate to create upper and lower chambers. We then added 600 μl medium with 10% fetal bovine serum (FBS) to the lower chamber and 200 μl serum-free conditioned medium with 2 × 10^4^ cells to the upper chamber. A cotton swab was used to wipe the upper chamber cells, and invasion cells were fixed and stained with crystal violet for counting after 24 h.

### Statistical analysis

Multivariate data analysis was conducted using Cox proportional hazard model and Kaplan–Meier method for calculating survival curves and comparing them with the log-rank test. R (version 3.6) software was used for all statistical analyses. Statistical significance was determined by a p-value <0.05 on both sides of the test.

## Results

### Immune subtype analysis showed the two immune groups of RCC

**Figure 1:**
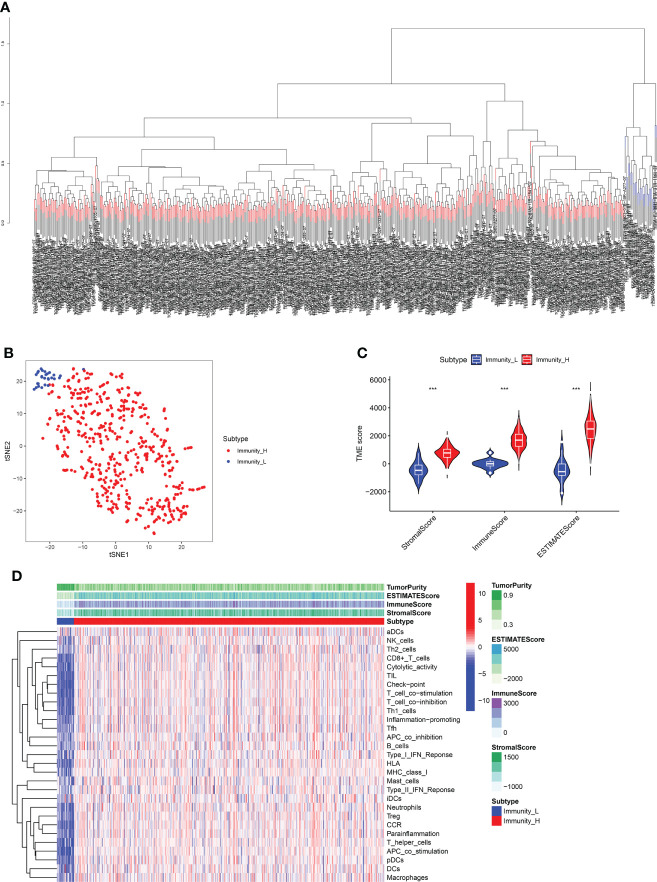
**(A)** The ssGSEA method cluster the TCGA dataset containing RCC samples based on the expression profiles of the immune cells. **(B)** Dimensionality reduction algorithm t-SNE showed that the subtypes were highly consistent with two-dimensional t-SNE distribution patterns. **(C)** TMB score analysis between immune_H and immune_L groups. **(D)** The heatmap demonstrated the different expression level of immune cells between immune_H and immune_L groups *** P < 0.001.

In this study, 29 immune-related genomes representing various immune cells, pathways, and functions were assessed. ssGSEA method was used to cluster TCGA dataset containing RCC samples based on the immune cells’ expression profiles (**Figure 1A**). Dimensionality reduction algorithm t-SNE showed that the subtypes were highly consistent with two-dimensional t-SNE distribution patterns at a k-value of 2 (**Figure 1B**). A group with high immunity was known as Immunity_H, while a group with low immunity was known as Immunity_L. Among Immunity_H subtypes, immune cell infiltration and immune pathways activation were reported, which indicates an immune hot phenotype. According to the Immunity_L subtype, there was low immune cell infiltration, indicating a cold immune phenotype.

Moreover, the immune subtype analysis demonstrated that each was associated with the different immune components in RCC patients. The Immunity_H subtype showed a higher stromal score, immune score, and estimate score than the Immunity_L subtype (**Figure 1C**). The heatmap demonstrated that Immunity_L subtype was enriched with more tumor purity than Immunity_H subtype. Immunity_H subtype had greater infiltration and immune pathways activation than Immunity_L subtype in terms of immune cell infiltration and immune pathways activated (**Figure 1D**).

### Differences in human leukocyte antigen genes, immune-related genes, immune cell infiltration, and activated immune pathways related to immune subtype

**Figure 2:**
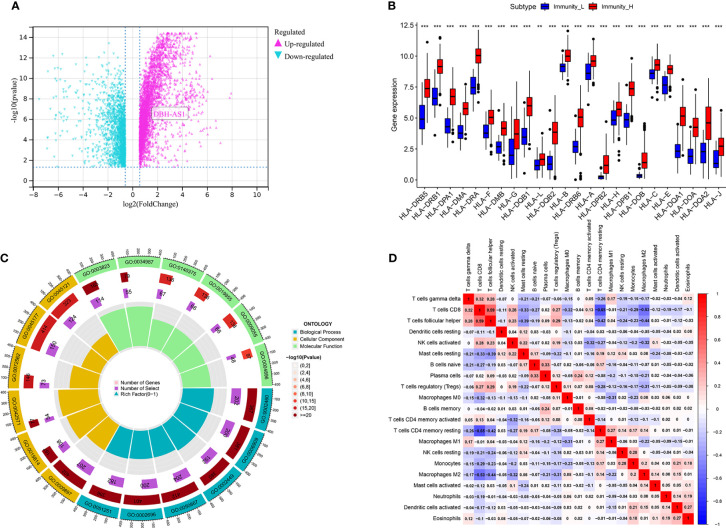
**(A)** The volcano map demonstrates the different expression genes between immune_H and immune_L groups; **(B)** the different expression level of 24 genes encoding human leukocyte antigens between immune_H and immune_L groups. **(C)** GO enrichment analysis based of different immune subtypes. **(D)** The correlation analysis between different immune cells *** P < 0.001; ** P < 0.01.

Furthermore, we identified differences in gene expression between Immunity_H and Immunity_L. Based on the results, 2,819 genes were upregulated in the Immunity_H group as opposed to the Immunity_L group. Compared to the Immunity_L group, 1,847 genes were downregulated in the Immunity_H group (**Figure 2A**). Immune surveillance is facilitated by presenting tumor-associated antigens by MHC class I complexes. Immunotherapies that target immune checkpoints benefit from this presentation. Our study examined 24 genes encoding human leukocyte antigens (HLA). A significant reduction in immunological HLA gene expression was observed in Immunity_L, suggesting that tumor cells evade immune surveillance by presenting antigens in a compromised manner (**Figure 2B**). GO enrichment analysis revealed that several genes involved in Immunity_H were associated with lymphocyte-mediated immunity, positive cell activation regulation, immune-dependent cell death, positive regulation of leukocyte activation, positive regulation of lymphocyte activation, B cell-mediated immunity, immunoglobulin-mediated immune response, and immune response-activating cell surface receptor signaling pathway, suggesting that this subtype is closely related to the immunotherapy (**Figure 2C**).

### Correlation analysis of antigen-presenting cells

**Figure 3:**
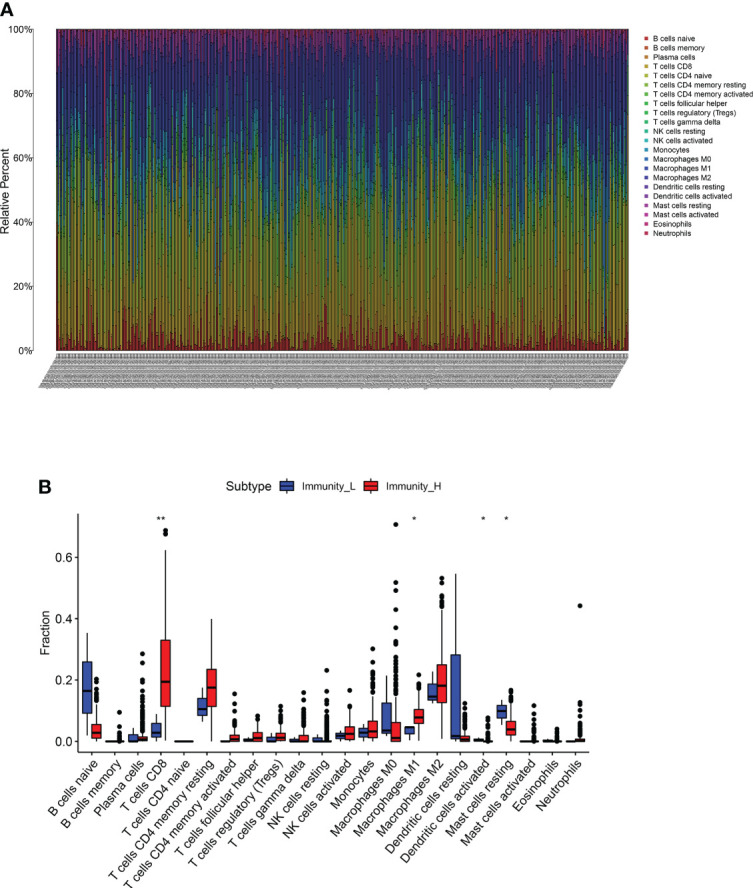
**(A, B)** The different expression of immune cells between immune_H and immune_L groups ** P < 0.01; * P < 0.05.

Further analysis of hub genes was performed with TIMER. **Figure 2D** demonstrates the internal correlation between different immune cells. Genes involved in Immunity_H were significantly upregulated in CD8+ T Cells and M1 macrophages. Genes involved in Immunity_L were significantly upregulated in activated dendritic cells and resting mast cells. These findings indicate that the genes involved in different immune subtypes have potential immunostimulatory effects. The genes promote the processing and presentation of immune cells by antigen-presenting cells to induce tumor response (**Figures 3A, B**).

### Immune gene coexpression profile in RCC cohort

**Figure 4:**
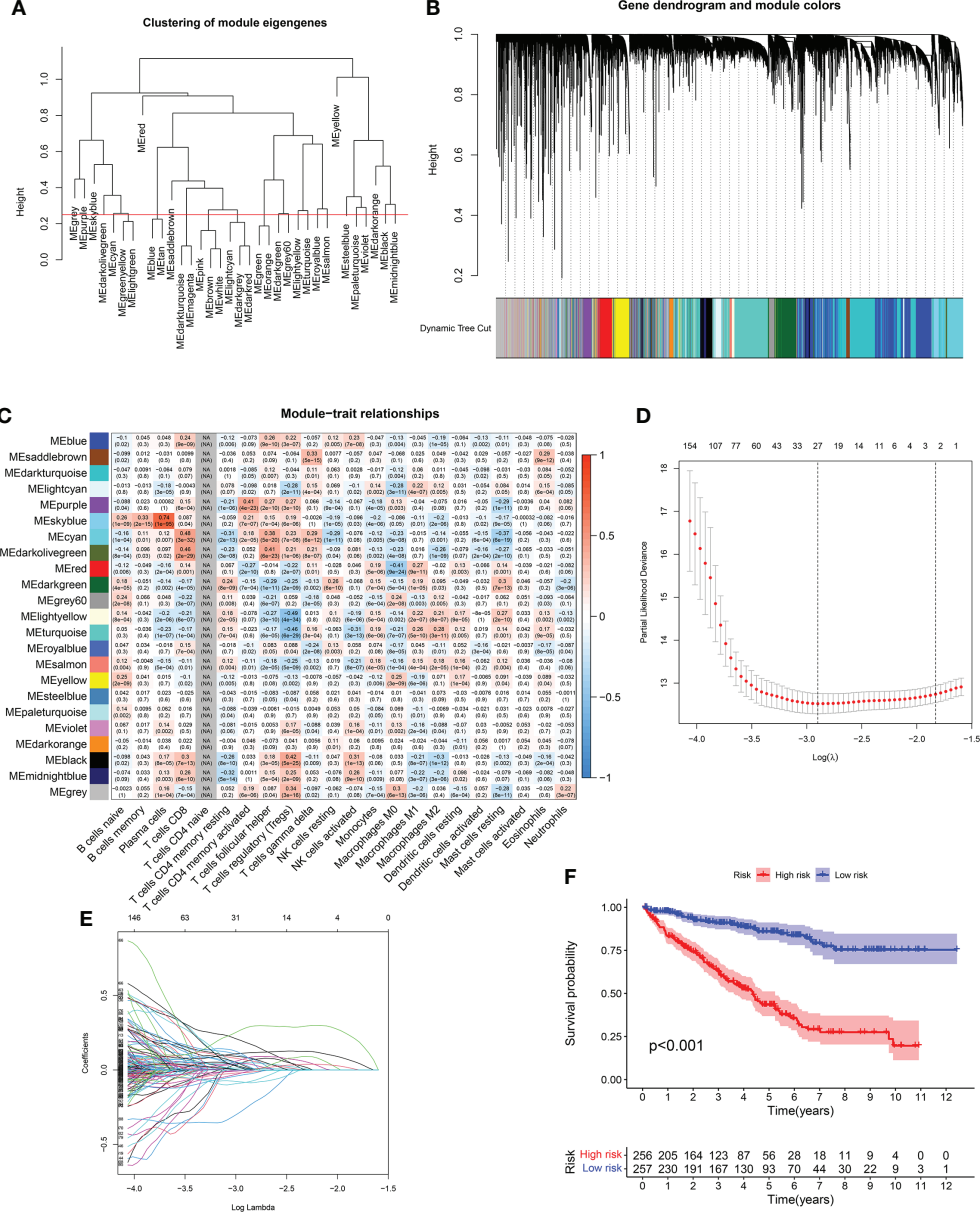
**(A, B)** WGCNA analysis of RCC cohort based on immune-related gene coexpression networks. **(C)** A total of 23 gene modules were identified based on WGCNA analysis of RCC cohort. **(D, E)** The LASSO regression analysis demonstrated that 27 immune-related genes were closely related to the prognosis of RCC patients. **(F)** The survival analysis between high- and low-risk groups.

Based on WGCNA analysis of the RCC cohort, immune-related gene coexpression networks were generated. As the clinical characteristics of the samples were immunoglobulins, the WGCNA network was constructed to explore potential biomarkers. A soft threshold of 9 was determined by the function “sft$powerEstimate” (**Figures 4A, B**). A total of 23 gene modules were identified based on TOM matrix, namely, cyan (n = 1,218), dark green (n = 1,463), dark olive green (n = 65), dark orange (n = 238), dark turquoise (n = 3,233), gray (n = 3,362), gray 60 (n = 350), light cyan (n = 890), light yellow (n = 312), midnight blue (n = 376), black (n = 914), blue (n = 2696), pale turquoise (n = 93), purple (n = 520), red (n = 941), royal blue (n = 308), saddle brown (n = 205), salmon (n = 457), sky blue (n = 208), steel blue (n = 129), turquoise (n = 2,912), violet (n = 80), and yellow (n = 1,005) modules (**Figure 4C**). Further module analysis and traits revealed that blue modules were highly correlated with immune cells (p-value < 0.01). Therefore, blue was selected for subsequent validation and analysis. There was a significant difference between the expression levels of the 23 modules’ characteristic genes and multiple immune cells.

### Construction of the prognosis-related predictive models based on immune subtype analysis

We obtained clinical information from RCC patients to further explore key genes in the candidate gene set. Then, we performed univariate and multivariate Cox and LASSO regressions to identify the genes associated with RCC. Based on univariate Cox regression results (p-value < 0.01), 2,033 genes associated with prognosis were identified. Then, we screened 27 prognosis-related genes (p-value < 0.01) by LASSO regression analysis (**Figures 4D, E**). Finally, we obtained the best risk score (risk score = AL161669.1 × -0.250430734267225 + BCL3 × 0.0337407354902806 + AC138649.2 × 0.401250063642218 + DBH-AS1 × 0.204477476109481 + NARF × 0.178383885858721 + AC002456.1 × 0.407989595839647 + DONSON × 0.19222775376306 + RTKN × -0.0510580913817709 + AC068338.3 × -0.720995150501497+ ZFYVE19 × -0.19065271282695 + AC069200.1 × 0.363732217837645 + TM4SF19-AS1 × 0.59294311636509 + UPK3B × 0.233561624709354) value for subsequent analysis by multivariate Cox regression. A Kaplan–Meier curve was used to analyze risk scores and divide patients into high- and low-risk groups. There was a significant difference in OS between the high- and low-risk groups for RCC (**Figure 4F**).

### Relationship between prognosis-related predictive models, immune cells, immune checkpoints, immunotherapy, and tumor microenvironment

**Figure 5:**
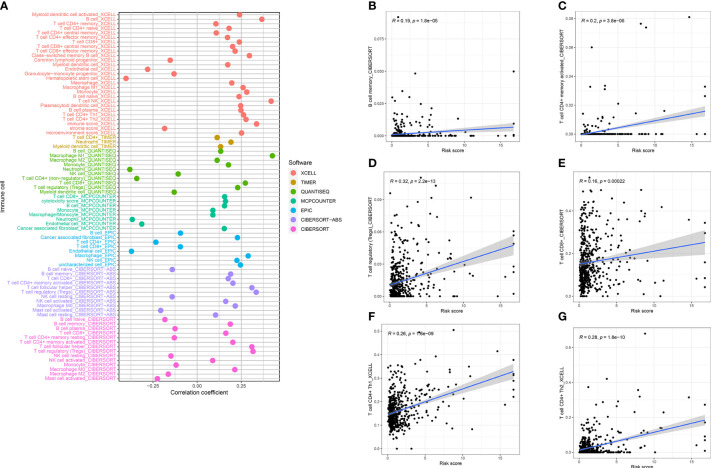
**(A)** Tumor microenvironment analysis demonstrated that risk score was closely related to B memory cells **(B)**, CD8+ T memory cells **(C)**, Tregs **(D)**, CD8+ T cells **(E)**, Th1 **(F)**, and Th2 **(G)**.

**Figure 6:**
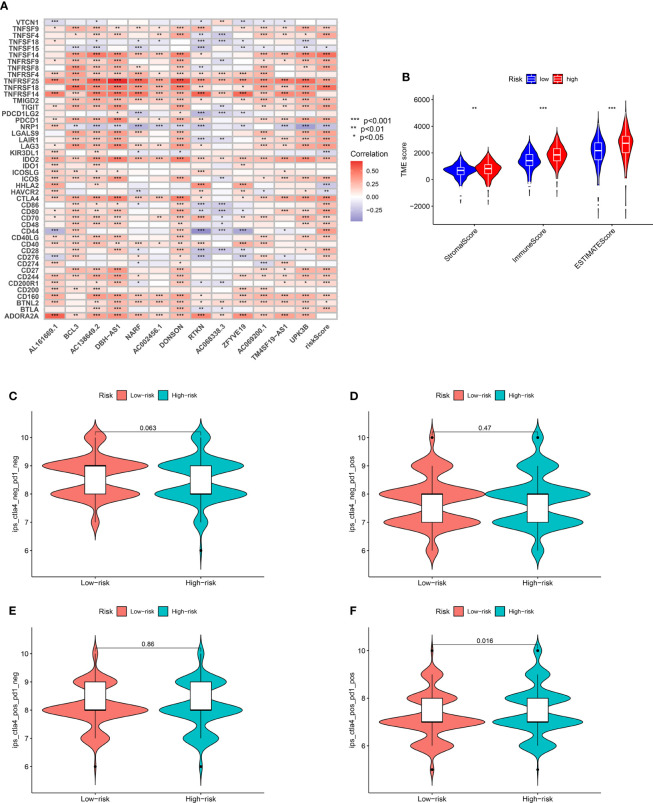
**(A)** Significant differences identified in the expression levels between immune checkpoint subtypes and prognostic-related genes. **(B)** TMB score analysis between high- and low-risk groups. **(C)** Immunotherapy evaluation of CTLA4 negative and PD1 negative patients between low- and high-risk groups. **(D)** Immunotherapy evaluation of CTLA4 negative and PD1 positive patients between low- and high-risk groups. **(E)** Immunotherapy evaluation of CTLA4 positive and PD1 negative patients between low- and high-risk groups. **(F)** Immunotherapy evaluation of CTLA4 positive and PD1 positive patients between low- and high-risk groups. *** P < 0.001; ** P < 0.01; * P < 0.05.

Several cell types within the tumor microenvironment, including fibroblasts, immune cells, extracellular matrix, growth factors, inflammatory mediators, and cancer cells, have different properties and chemical characteristics. Diagnosis, survival outcomes, and drug sensitivity strongly influence the tumor microenvironment. There was a significant difference in memory B-cell counts, CD4+ T memory cell counts, and CD4+ Th2 cell counts between the two groups with different risk scores (**Figures 5A–G**). There was a comparison of immune checkpoint-related gene expression levels among different risk groups. Significant differences were seen in expression levels between immune checkpoint subtypes for several genes related to immune checkpoints. Based on these results, there were differences in immune regulatory pathways between different risk groups. Immune response dysregulation can lead to different prognoses in the two groups of patients (**Figure 6A**). In RCC patients, the tumor microenvironment significantly affects survival outcomes (**Figure 6B**).

**Figure 7:**
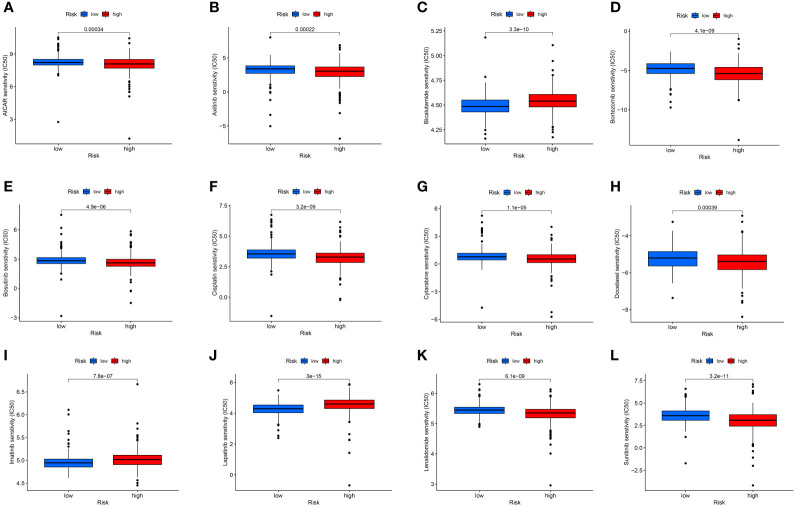
The chemotherapy sensitivity of aicar **(A)**, axitinib **(B)**, bicalutamide **(C)**, bortezomib **(D)**, bosutinib **(E)**, cisplatin **(F)**, cytarabine **(G)**, docetaxel **(H)**, imatinib **(I)**, lapatinib **(J)**, lenalidomide **(K)**, and sunitinib **(L)**.

Additionally, immunotherapy using CTLA4-negative and PD1-positive patients showed promise in high-risk groups (**Figures 6C–F**). Therefore, surgery in early RCC in conjunction with chemotherapy has proven to be a promising treatment. Furthermore, the chemotherapy sensitivity of the tumor sample was predicted using GDSC database and “pRRophetic” R package. All prognosis-related predictive models were found to have significant associations with aicar, axitinib, bicalutamide, bortezomib, bosutinib, and cisplatin sensitivity, and cytarabine, docetaxel, imatinib, lapatinib, lenalidomide, and sunitinib (**Figures 7A–L**).

### Functional enrichment of immune gene coexpression modules

**Figure 8:**
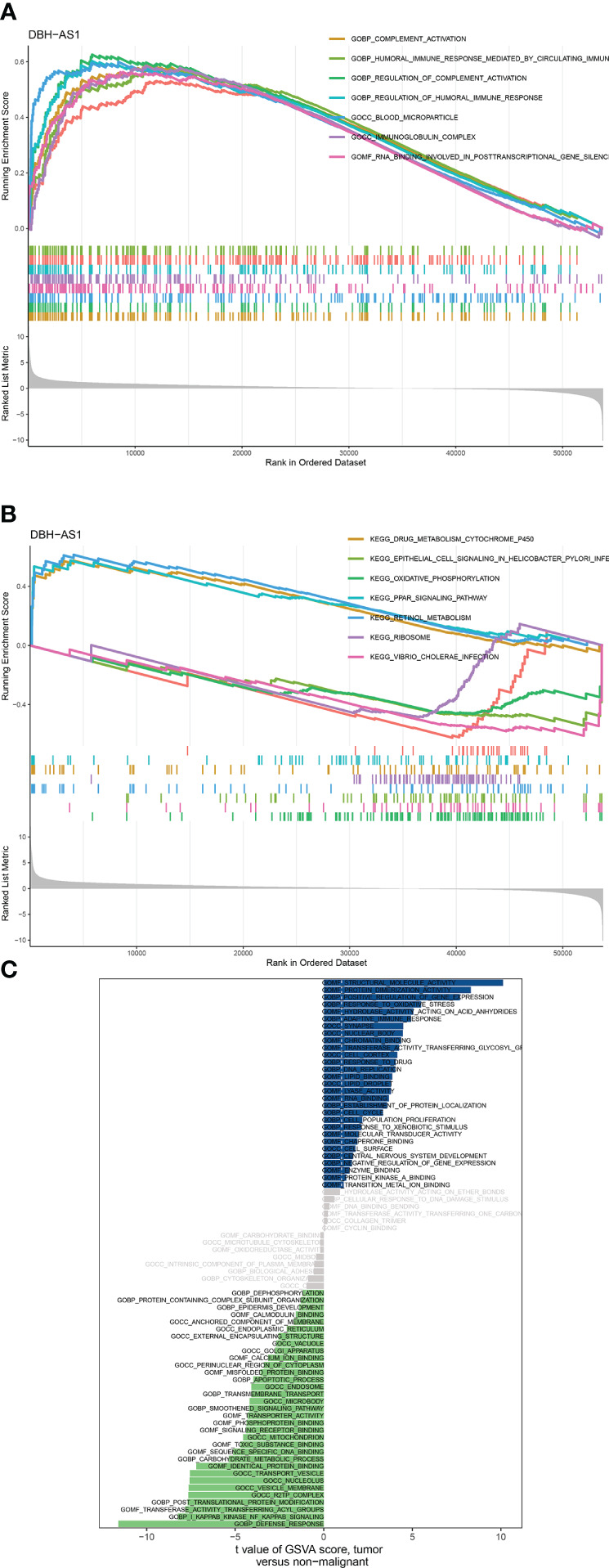
**(A, B)** GSEA analysis between DBH-AS1 high- and low-expression groups. **(C)** GSVA analysis between DBH-AS1 high- and low-expression groups.

According to the differential expression analysis and prognostic prediction model, DBH-AS1 may be a key gene in RCCs. Consequently, we performed a GSEA and GSVA enrichment analysis on DBH-AS1. GSEA enrichment analysis indicated that DBH-AS1 was significantly enriched in various pathways. GSEA enrichment analysis showed that genes were enriched in pathways involved in complement activation, complement activation regulation, blood microparticle, RNA binding involved in posttranscriptional gene silencing, immunoglobulin complex, humoral immune response regulation, B-cell-mediated immunity, humoral immune response mediated by circulating immunoglobulin, and humoral immune response. Furthermore, the specific signaling pathways associated with the DBH-AS1 were investigated, and potential molecular mechanisms involved in the RCC pathogenesis and progression were investigated (**Figures 8A, B**). According to GSVA, the differential pathways between DBH-AS1 expression groups were mainly signaling pathways such as adaptive immune response, apoptotic process, biological adhesion, carbohydrate metabolic process, cell cycle, cell population proliferation, cellular response to DNA damage stimulus, central nervous system development, and cytoskeleton organization (**Figure 8C**).

### DBH-AS1 evaluation in the renal carcinoma cells

**Figure 9:**
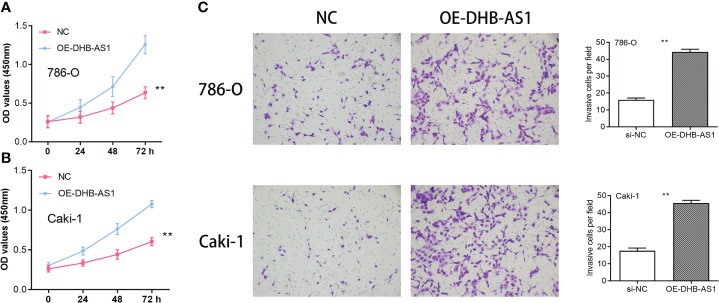
**(A, B)** Cell Counting Kit-8 Assay in 786-O and Caki-1 cells. **(C)** Transwell assay in 786-O and Caki-1 cells ** P < 0.01.

According to studies using CCK8, the proliferative capacity of 786-O and Caki-1 cells overexpressing DBH-AS1 was higher than that of cells transfected with a vector that was empty (**Figures 9A, B**). In addition, the transwell assay of cell invasion revealed that 786-O and Caki-1 cells overexpressing DBH-AS1 showed higher invasion capacity compared with empty vector (**Figure 9C**).

## Discussion

RCC is among the 10 most common cancers worldwide, with men being more likely to be affected (16). It is the seventh most common cancer among men, and the 12th most common cancer among women, accounting for 2.6% of all cancers in the United States (17). RCC is, therefore, a serious health threat to humans and a heavy economic burden. In addition to surgery and radiation therapy (RT), ablation therapy, chemotherapy, and emerging immunotherapies can be used to treat RCC (18). Over the past decade, there have been many changes in RCC treatment landscape as new treatments have altered the efficacy of conventional treatments efficacy (19). The immunotherapy goal is to enhance anti-tumor immune responses through immune system activation. This therapy has revolutionized cancer treatment by enhancing anti-tumor immune responses. However, immunotherapy may not have a significant effect on RCC prognosis. Although RCC is a known immunogenic disease, it can evade the immune system by downregulating human leukocyte antigen class I (20). Due to Fas ligand expression, antigen presentation becomes ineffective because T cells undergo apoptosis, and immune suppressants are secreted. Currently, a clinical trial is investigating immunotherapy efficacy in RCC patients (21).

According to ssGSEA analysis, RCC cohort patients were divided into Immunity_H and Immunity_L. Our results showed that many immune-related genes were considered DEGs after performing the differential expression analysis between Immunity_H and Immunity_L groups. Furthermore, DEGs are associated with enrichment pathways. A GO enrichment analysis revealed that several immune-related pathways, including lymphocyte-mediated immunity, B-cell-mediated immunity, immunoglobulin-mediated immune response, and immune-response-activating cell surface receptor signaling pathway, were closely related to different immune subtypes. The HLA class I molecule is involved in recognizing, presenting, and lysing tumor cells by cytotoxic T lymphocytes (CTLs), and their defects may facilitate tumor immune escape (22). Studies indicated that RCC patients treated with TKIs whose HLA class I expression is downregulated have a lower response rate and a worse prognosis. HLA class I expression correlates with tumor CTL infiltration and function (23). According to our study, the Immunity_L group had significantly reduced expression of most immune HLA genes, suggesting that impaired antigen presentation on tumor cells may contribute to cancer development.

We explored the genes correlated with RCC prognosis in further analysis. Based on 13 immune-related genes, we built a prognostic prediction model using univariate Cox regression, multivariate Cox regression, and lasso regression, including AL161669.1, BCL3, AC138649.2, DBH-AS1, NARF, AC002456.1, DONSON, RTKN, AC068338.3, ZFYVE19, AC069200.1, TM4SF19-AS1, and UPK3B. In the survival outcomes analysis, low-risk subgroups outlived patients in high-risk subgroups. This indicates that immunotype is a potential prognostic biomarker for RCC patients. The tumor microenvironment (TME) is a complex structure composed of tumor cells, non-malignant cells, blood vessels, extracellular matrix, and other substances. These different immune cell types play key regulatory roles in tumors. It has been reported that TME characteristics can be used as markers for evaluating tumor cell responses to immune therapy and can influence the clinical outcome of cancer patients. This study showed that immune cells such as memory B cells, CD4+ T memory cells, T regulatory cells, CD8+ T cells, CD4+ Th1 cells, CD4+ Th2 cells, and other microbes in tumors were implicated in tumor growth and invasion. Additionally, the two groups immune-related characteristics may reflect underlying mechanisms that regulate tumor immune response and escape, which explains the different survival rates. According to studies, immune checkpoint blockade treatment (ICB) is only beneficial for some patients, and the molecular characteristics of TME are closely related to radiotherapy and chemotherapy.

We found that DBH-AS1 may play a key role in the immunotherapy of RCC patients based on differential expression analysis and prognostic prediction models. The previous study discovered that DBH-AS1 is involved in many tumors. According to Ye et al., DBH-AS1 regulates the growth of pancreatic cancer and is a viable target for predicting gemcitabine responses in patients with pancreatic cancer (24). DBH-AS1 promotes hepatocellular carcinoma development through miR-138/FAK/Src/ERK pathways in hepatocellular carcinoma as well (25). GSEA and GSVA analyses were performed in this study to investigate potential pathways involved in DBH-AS1. Moreover, they demonstrated the association between DBH-AS1 and several immune-related pathways, including immunoglobulin complex, humoral immune response regulation, B-cell-mediated immunity, humoral immune response mediated by circulating immunoglobulin, and humoral immune response.

The present study showed that immune subtypes were significantly associated with immunotherapy drug sensitivity, including aicar, axitinib, bicalutamide, bortezomib, bosutinib, cisplatin, cytarabine, docetaxel, imatinib, lapatinib, lenalidomide, and sunitinib. Due to the restricted therapeutic alternatives and clinical benefits of chemotherapy, seeking more effective treatment methods was imperative. As tumor-specific and non-specific antigens are expressed in cancerous cells, immunotherapy represents a promising new approach to treating malignancies. In recent decades, treatment options for RCC patients have expanded rapidly, and targeted immunotherapy is now one of the most effective treatments. Several emerging drugs are currently being tested in clinical trials to boost anti-tumor immune responses. As more treatment options are available, it is essential to develop biomarkers that can help stratify patients and determine the best options for them and the treatment sequence that will overcome resistance to the treatments.

## Conclusion

The immune-related genes (DBH-AS1) identified in the present study are potential targets for immunotherapy development against RCC. This study provides a theoretical basis for developing RCC immunotherapy. Furthermore, the immune subtypes can be used to explore strategies for improving immunotherapy response in RCC patients.

## Data availability statement

The datasets presented in this study can be found in online repositories. The names of the repository/repositories and accession number(s) can be found in the article/supplementary material.

## Author contributions

XC, TZ, XZ, CL and MT collected the data and performed the analysis. MG, DX and ZW wrote the manuscript. All authors contributed to the article and approved the submitted version.

## Conflict of interest

The authors declare that the research was conducted in the absence of any commercial or financial relationships that could be construed as a potential conflict of interest.

## Publisher’s note

All claims expressed in this article are solely those of the authors and do not necessarily represent those of their affiliated organizations, or those of the publisher, the editors and the reviewers. Any product that may be evaluated in this article, or claim that may be made by its manufacturer, is not guaranteed or endorsed by the publisher.
